# A case of simultaneous abdominal wall metastasis of hepatocellular carcinoma with long-term relapse-free survival after laparoscopic resection

**DOI:** 10.1007/s13691-021-00484-0

**Published:** 2021-04-18

**Authors:** Michitoshi Goto, Kazuhiro Sakamoto, Shingo Kawano, Shinya Munakata, Masaya Kawai, Shun Ishiyama, Kiichi Sugimoto, Makoto Takahashi, Yutaka Kojima, Natsumi Tomita, Harumi Saeki, Shuichiro Shiina

**Affiliations:** 1 Department of Coloproctological Surgery, Faculty of Medicine, Juntendo University, 2-1-1 Hongo, Bunkyo-ku, Tokyo, 113-8421 Japan; 2 Department of Gastroenterological Surgery, Faculty of Medicine, Juntendo University, 2-1-1 Hongo, Bunkyo-ku, Tokyo, 113-8421 Japan; 3 Department of Human Pathology, Faculty of Medicine, Juntendo University, 2-1-1 Hongo, Bunkyo-ku, Tokyo, 113-8421 Japan; 4 Department of Gastroenterology, Faculty of Medicine, Juntendo University, 2-1-1 Hongo, Bunkyo-ku, Tokyo, 113-8421 Japan

**Keywords:** HCC (hepatocellular carcinoma), Extrahepatic metastasis, Laparoscopic surgery (resection), Radiofrequency ablation (RFA)

## Abstract

We report our experience of an extremely rare case of a simultaneous extrahepatic metastasis of hepatocellular carcinoma (HCC) with long-term relapse-free survival, treated by laparoscopic resection of an abdominal wall tumor and subsequent radiofrequency ablation (RFA) of an intrahepatic lesion. A 76-year-old man visited a local clinic for right lower abdominal pain. He was treated with antibiotics and the symptom resolved. However, a mass was detected in the same region and he was referred to our hospital for further evaluation. Computed tomography (CT) of the abdomen showed a mass 5 cm in diameter, raising suspicions of an intra-abdominal tumor. Laparoscopic surgery was performed, and the tumor was found in the abdominal wall and completely resected. Histopathological examination yielded a diagnosis of extrahepatic HCC. Post-operative positron emission tomography (PET)-CT showed increased uptake of fluorodeoxyglucose in segment 3 (S3) of the liver. On performing a liver biopsy, HCC was diagnosed. Subsequently, the S3 lesion was treated with radiofrequency ablation. The patient has remained relapse-free for 6 years without further treatments.

## Introduction

Extrahepatic metastasis of hepatocellular carcinoma (HCC) is present at the time of diagnosis in 5–15% of HCC patients [1, 2]. Extrahepatic metastases of HCC are commonly detected in lung tissue, lymph nodes, and bones [3, 4]. The standard treatment for extrahepatic metastasis of HCC has not been established. Furthermore, patients with simultaneous metastasis for HCC have a poor prognosis [5]. We experienced a case of an HCC with simultaneous extrahepatic metastasis treated by laparoscopic resection, and a primary liver lesion subsequently treated with radiofrequency ablation (RFA) after diagnosis based on the post-operative positron emission tomography (PET)—computed tomography (CT) image.

## Case report

**Fig. 1:**
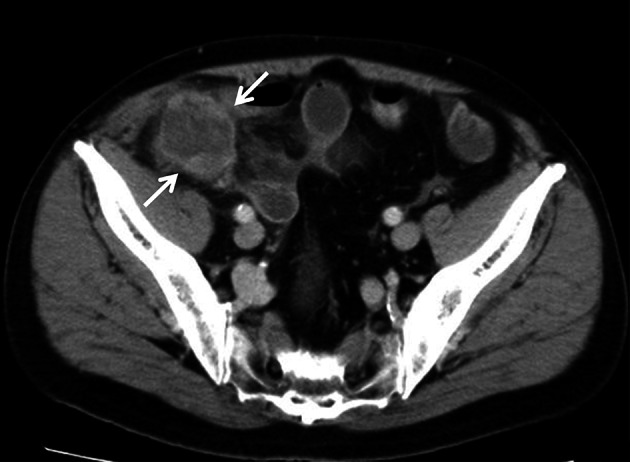
Abdominal CT. Abdominal CT revealed a mass with peripheral enhancement approximately 5 cm in diameter (arrow)

A 76-year-old man visited a local clinic with the chief complaint of right lower abdominal pain. The symptom improved with the administration of antibiotics and anti-inflammatory agents. However, a mass was detected in the same region and he was referred to our hospital. On physical examination, the abdomen was soft and flat without tenderness. A mass slightly larger than a chicken egg was palpable in the right lower abdomen. No medical history of abdominal surgery such as appendectomy. The blood test results showed no evidence of hepatic dysfunction or increased inflammatory reactions. The levels of the following tumor markers were normal: carcinoembryonic antigen (CEA), 0.5 ng/mL; cancer antigen 19-9 (CA19-9), 10 ng/mL; alpha-fetoprotein (AFP), 4 ng/mL; and protein induced by vitamin-K absence-II (PIVKA-II), 22 mAU/mL. He had negative results for both hepatitis B surface antigen and hepatitis C antibody tests. The CT of the abdomen revealed a mass in the right lower abdomen. The diameter of the mass was 5 cm, with an abnormal internal low density and peripheral enhancement (Fig. 1). Magnetic Resonance Imaging (MRI) of the abdomen revealed a mass non-uniform low intensity on T2-weighted images. In the T1-weighted image, an isointensity to that of muscle and a faint high intensity were mixed. CT and MRI showed that the tumor was in contact with the small intestine and the parietal peritoneum, and a fat layer was confirmed between the tumor and the iliopsoas muscle. An upper and lower gastrointestinal endoscope was carried out, but there were no abnormal views. The preoperative diagnosis was a mesenteric tumor, and gastrointestinal stromal tumor (GIST) was suspected. Based on the above results, we performed laparoscopic surgery including further diagnosis and treatment.

**Fig. 2:**
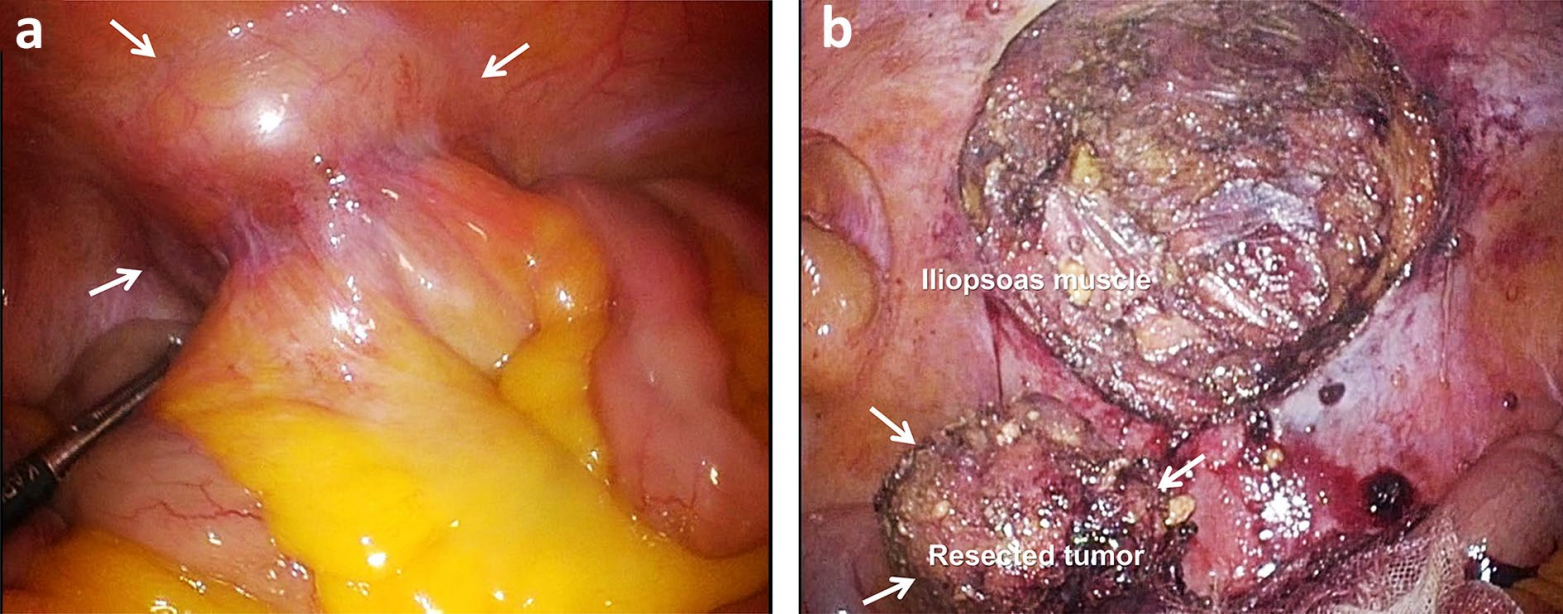
Laparoscopic findings. **a** A hemispheric, protruding tumor adherent to the mesentery of the small intestine was detected in the right lower abdominal wall (arrows). **b** An incision was made in the peritoneum, and the oblique muscle was partially scraped and resected. The tumor was completely resected macroscopically (arrows)

A laparoscopic port was inserted into the umbilicus. Additional ports were inserted in the right upper abdomen, left upper abdomen, and lower abdomen. A total of four ports were used for the surgery. A hemispheric, protruding tumor adherent to the mesentery of the small intestine was detected in the right lower abdominal wall (Fig. 2a). The mesentery was detached and the tumor was found to be covered by the peritoneum. We diagnosed that the tumor was extraperitoneal. An incision was made in the peritoneum, and the iliopsoas muscle was partially scraped and resected in order to remove the tumor completely, but there was no clearly infiltration into the muscle. The tumor was completely resected macroscopically (Fig. 2b), put in a bag, and extracted through the umbilical port, which was extended by 5 cm. The operation time was 140 min and blood loss was 30 mL. The patient’s post-operative course was uneventful and he was discharged from the hospital on day 7 post-surgery.

**Fig. 3:**
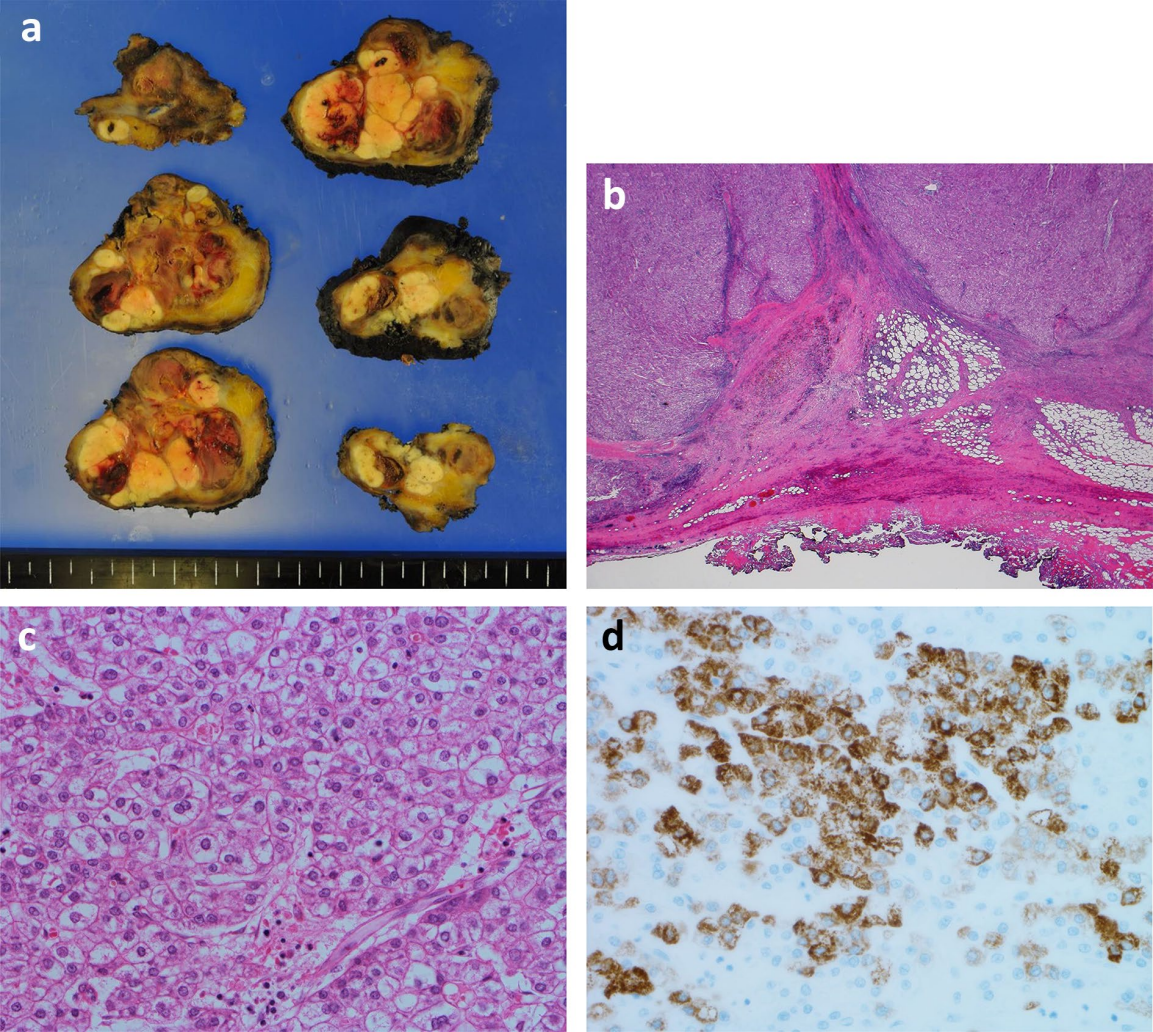
Histological examination. **a** A tumor measuring 55 × 45 × 34 mm in size. **b** Adipose tissue and muscle tissue are found around the tumor, and they are clearly separated by a fibrous capsule. (H.E.stain × 10) **c** Cells with clear to eosinophilic vesicles show a cord-like structure consisting of several layers and proliferate. (H.E.stain × 400). **d** The immunochemistry specimen was positive. (Hepatocyte paraffin antigen-1 stain × 400)

Histopathology revealed a tumor measuring 55 × 45 × 34 mm in size. Adipose tissue and muscle tissue are found around the tumor, and they are clearly separated by a fibrous capsule (Fig. 3a, b). The specimen stained with hematoxylin and eosin showed that the cells exhibiting eosinophilic cytoplasm were arranged in trabeculae and proliferated, leading to a diagnosis of extrahepatic HCC.Well-differentiated to moderately-differentiated tumor with no normal hepatocytes (Fig. 3c). The immunochemistry specimen was positive for hepatocyte paraffin antigen-1 (Fig. 3d).

**Fig. 4:**
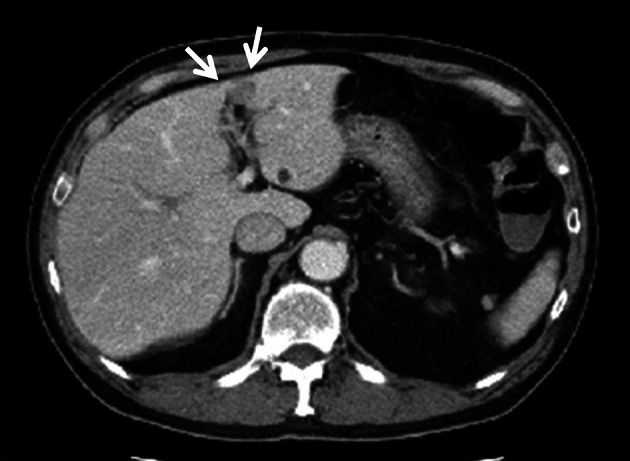
Abdominal CT. The preoperative CT revealed a slightly low density area of 17 mm in diameter was detected in segment 3 of the liver (arrows)

**Fig. 5:**
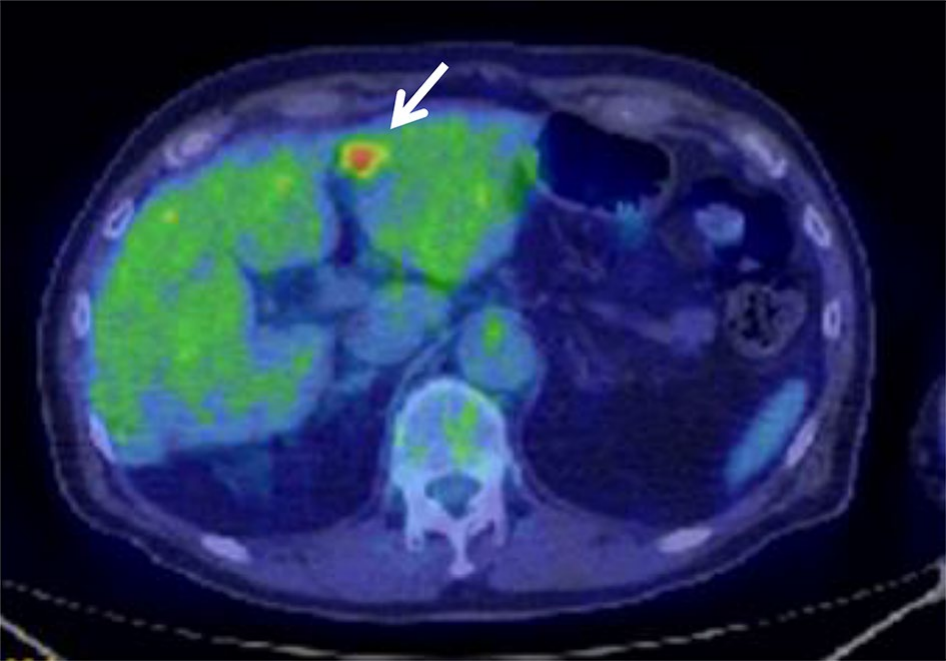
PET-CT. Post-operative PET-CT revealed an increased uptake (SUVmax 15.3) in the liver (arrow)

**Fig. 6:**
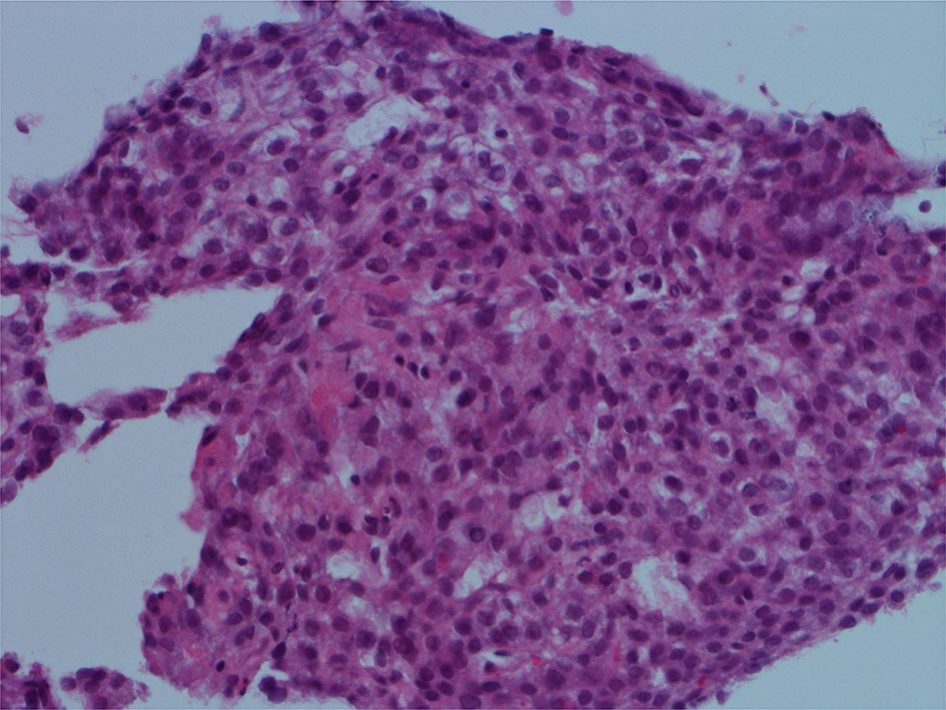
Histological examination by biopsy. Moderately differentiated hepatocellular carcinoma

After the resected abdominal wall tumor was pathologically diagnosed as extrahepatic HCC, the CT image of the abdomen taken preoperatively was reviewed and a slightly-low-density area of 17 mm in diameter was detected in segment 3 (S3) of the liver (Fig. 4). The PET- CT, MRI and Abdominal ultrasound (US) were performed after surgery considering that there are accessory lesions in the liver and other parts. The PET- CT image revealed increased uptake of fluorodeoxyglucose (with a maximum standardized uptake value of 15.3) only in the same site (Fig. 5). MRI and US also revealed lesions in the same site. Therefore, I thought that it was a single HCC for only S3 of the liver. Liver biopsy yielded a diagnosis of HCC (Fig. 6). We recommended surgical liver tumor resection, but patients preferred less invasive treatment and chose RFA. The patient underwent radiofrequency ablation (RFA). It was carried out on a regular basis observation by CT and MRI, but the patient has remained relapse-free for 6 years without further treatment.

## Discussion

Common intra-abdominal tumors include carcinomas, mesenchymal tumors, and lymphomas. They are localized to various regions including the gastrointestinal tract, solid organs, mesentery, and abdominal wall. Laparoscopic surgery is beneficial because it provides a magnified view for confirming tumor localization and invasion of adjacent organs, and allows for complete resection in some cases [6]. Although our patient was preoperatively diagnosed with a mesenteric tumor, he was diagnosed with a tumor of the abdominal wall during laparoscopic surgery. The tumor margin was clearly confirmed, enabling complete resection. Thus, the benefits of laparoscopic surgery were confirmed.

In our case, an abdominal wall mass was pathologically diagnosed with an extrahepatic HCC. Malignant transformation of ectopic liver and metastasis of intrahepatic HCC were considered to be the pathogenetic mechanisms in this case. The ectopic liver is an extremely rare congenital malformation with an incidence of 0.24–0.86% [7, 8]. Ectopic liver tissue is most commonly found in the gallbladder. In one reported case, the ectopic liver tissue attached to the gallbladder was detected during cholecystectomy for cholelithiasis and resected [9]. Other common sites include the greater omentum, adrenal gland, round ligament of the liver, and spleen, that is, regions around the liver [7, 8]. Therefore, malignant transformation of the ectopic liver was unlikely in this case. Since HCC was detected in S3 of the liver by post-operative examinations, it was reasonable to consider that the tumor of the abdominal wall was a simultaneous extrahepatic metastasis of the HCC.

Extrahepatic metastasis of HCC is found in 5–15% of HCC patients [1, 2], and is most commonly detected in the lung, lymph nodes, and bones [3, 4]. Our case was considered peritoneal dissemination rather than lymph node metastasis than that there was no lymphoid tissue in the excised specimen. It was thought that the HCC that had disseminated to the peritoneum had grown as a mass outside the peritoneum and was considered to be very rare.

When extrahepatic metastasis is simultaneously detected, systemic therapy would be a reasonable first choice of treatment; however, standard treatments for extrahepatic metastasis of HCC have not been established.

The prognosis for HCC patients with extrahepatic metastasis is poor; however, Bauschke et al. [5] reported that patients with stage IV HCC had a significantly longer long-term survival after resection of the primary lesion and extrahepatic tumor, compared to those with local ablative therapy or systemic treatment. However, only nine patients (6.5%) of 138 patients with stage IV HCC underwent R0 resection (lymph nodes in 6, lung in 2, and omentum in 1), resulting in a better prognosis. Of the 6 lymph node resected patients, five patients had one positive lymph node and one patient had two positive nodes. Surgical resection is regarded as the only treatment that can potentially cure or extend survival in patients with extrahepatic oligometastasis of HCC. Xia et al. [4] reported that the prognosis in HCC patients with extrahepatic metastasis was significantly worse in patients with lymph node metastasis. When selecting a treatment method, it is necessary to consider the sites and the number of lesions of extrahepatic metastases. In our case, it was considered that there was no residual HCC due to surgery and RFA, so we decided to follow up closely.

In extrahepatic metastases of HCC, the metastasis of the abdominal wall is very rare without thoracoabdominal wall seeding tumor after ultrasound-guided interventional treatments for HCC [10, 11]. We searched PubMed for case reports describing intra-abdominal metastases of HCC using the following keywords: hepatocellular carcinoma, extrahepatic metastasis, intra-abdominal metastasis, and abdominal wall. We found two patients with abdominal wall metastases. There were metachronous metastases, and the two patients died due to liver failure, 6 months and 20 months after surgical resection, respectively [3]. Xia et al. [4] reported that peritoneal metastases/abdominal wall metastases were identified in seven patients out of 132 HCC patients with extrahepatic metastases. However it was not described in further details.

The patient has since presented no evidence of metastasis or recurrence to date, 6 years after treatment by laparoscopic resection of extrahepatic lesion and RFA of the intrahepatic lesion. Because, it can provide an opportunity for a good long-term prognosis that the patients had maintained good liver function without viral hepatitis, cirrhosis. In extrahepatic oligometastasis of HCC, such as our case, it is considered that surgical treatment may contribute a better prognosis for some patients.
